# Titrating the Smell of Fear: Initial Evidence for Dose-Invariant Behavioral, Physiological, and Neural Responses

**DOI:** 10.1177/0956797620970548

**Published:** 2021-03-22

**Authors:** Jasper H. B. de Groot, Peter A. Kirk, Jay A. Gottfried

**Affiliations:** 1 Department of Neurology, University of Pennsylvania; 2 Behavioural Science Institute, Radboud University; 3 Department of Experimental Psychology, University College London; 4 Institute of Cognitive Neuroscience, University College London; 5 Department of Psychology, University of Pennsylvania

**Keywords:** fear, olfaction, dose response, sweat, social cognition, functional MRI, amygdala, morph task, communication, sniff, open data, open materials

## Abstract

It is well accepted that emotional intensity scales with stimulus strength. Here, we used physiological and neuroimaging techniques to ask whether human body odor—which can convey salient social information—also induces dose-dependent effects on behavior, physiology, and neural responses. To test this, we first collected sweat from 36 males classified as low-, medium-, and high-fear responders. Next, in a double-blind within-subjects functional-MRI design, 31 women were exposed to three doses of fear-associated human chemosignals and neutral sweat while viewing face morphs varying between expressions of fear and disgust. Behaviorally, we found that all doses of fear-sweat volatiles biased participants toward perceiving fear in ambiguous morphs, a dose-invariant effect generally repeated across physiological and neural measures. Bayesian dose-response analysis indicated moderate evidence for the null hypothesis (except for the left amygdala), tentatively suggesting that the human olfactory system engages an all-or-none mechanism for tagging fear above a minimal threshold.

One striking capacity humans share with other animals is that our body odors can transmit social information (e.g., McGann, 2017; Parma et al., 2017; Roberts et al., 2020; cf. Wyatt, 2020, for a critical view). Physiological changes impact the quality (Smeets et al., 2020) and quantity of odor molecules emitted by senders, providing receivers with cues about the sender’s internal state. For example, experiments in the fields of psychology and neuroscience have yielded abundant support for the role of human fear odor in modulating human perception, behavior, and neural responses (for a meta-analysis, see de Groot & Smeets, 2017). Receivers exposed to sweat emitted by a fearful individual were typically quicker to perceive fearful facial expressions (Kamiloglu et al., 2018) and more likely to view ambiguous happy–fearful faces as fearful (Zhou & Chen, 2009). Exposure to fear odor has elicited adaptive fear-specific changes in facial musculature and concomitant increases in eye-scanning behavior and air intake to better detect threat (de Groot et al., 2012). Additionally, functional MRI (fMRI) studies have found that, compared with exercise sweat, fear-odor processing recruits the amygdala and fusiform face gyrus (FFG; Maier et al., 2019; Mujica-Parodi et al., 2009; Prehn-Kristensen et al., 2009), both of which are associated with the processing of fearful faces (Dolan et al., 2001). Subsequent imaging research has supported cross-modal integration by focusing on face processing in the context of fear odor, demonstrating greater engagement of FFG for the processing of fearful faces (Wudarczyk et al., 2016).

Earlier work has demonstrated robust cross-modal effects of fear odor by exclusively focusing on discrete emotion categories (fear vs. nonfear; e.g., de Groot & Smeets, 2017; Parma et al., 2017). A fearful person also emits qualitatively different molecules than happy and neutral individuals do (Smeets et al., 2020). Here, we examined another important aspect of emotions, namely, whether humans can chemically communicate emotion quantity or intensity. The fact that stimulus strength is proportionate to emotional intensity is well accepted: Sharper pins elicit more pain; louder growls elicit more fear. Humans can decode different emotional intensities in faces (Hess et al., 1997) and voices (Juslin & Laukka, 2001), yet evidence is lacking for such a phenomenon in the olfactory domain. Like other sensory systems, olfactory perception and behavior critically depend on maintaining stable representations of stimulus identity amid varying levels of odor intensity (Cleland et al., 2012; Roland et al., 2017; Wojcik & Sirotin, 2014). In this study, we focused on two related hypotheses: first, that each level of fear odor is capable of inducing fear-specific responses when compared with neutral, nonfearful sweat, and second, that increasing “doses” of fear in sweat would have a greater impact on the behavioral, physiological, and neural expression of fear.

Prior to testing hypotheses about the representation of fear-odor identity and intensity, we first began by obtaining fear and neutral sweat from a sender group. In this first stage (see de Groot et al., 2020), sweat was collected using absorbent pads under the armpits of 36 participants in whom fear and calmness were induced using prevalidated film clips of horror movies and nature movies, respectively. On the basis of subjective feelings and physiological measures (skin conductance, heart rate, breathing rate), we were able to sort the sender participants into three groups: those who experienced a low amount of fear as a result of watching the horror movie, those who experienced a medium amount of fear, and those who experienced a high amount of fear. By categorizing the senders in this way (de Groot et al., 2020), we found that fear intensity (low, medium, high) was linearly encoded in the quantity of sweat produced, with higher quantities of sweat also emitting more volatile odorant molecules, according to photoionization-detector measurements (Fig. 1; see also the Supplemental Material available online). These data supported the notion that humans may express the quantity of experienced fear in their sweat.

**Fig. 1.**
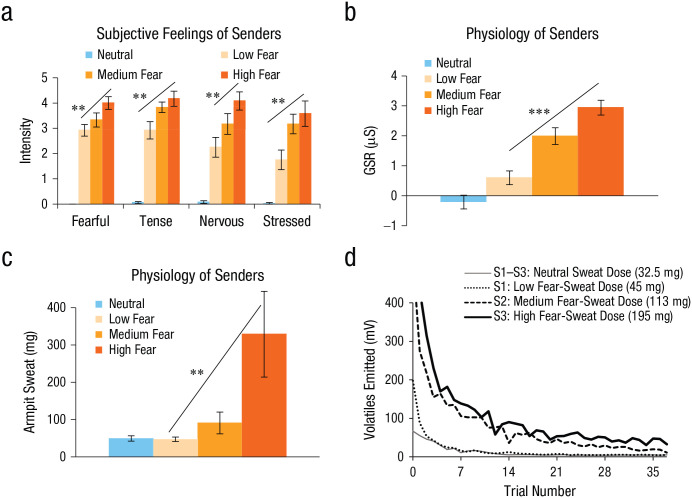
Experimental validation: classification of senders (*N* = 36) into equal groups of low-, medium-, and high-fear intensity (*n* = 12) on the basis of a regression-weighted combination of subjective and physiological responses. For each group (along with a neutral group), results are shown for (a) subjectively experienced fear, tension, nervousness, and stress; (b) galvanic skin response (GSR); and (c) quantity of armpit sweat. The quantity of volatile molecules emitted across time (as measured by a photoionization detector) is also shown (d) for three subjects (S1–S3) from pilot samples (see de Groot et al., 2020, for more details). Asterisks indicate significant differences among conditions (**p < .01, ***p < .001). Error bars represent ±1 *SE*.

## Method

Study materials and data are available on OSF at https://osf.io/y76p2/.

**Statement of Relevance**

Can human smells influence our social lives? There is evidence that psychological experience of fear translates into a unique chemical (odor) signature in that person’s sweat and that others can use the chemical signature as a cue to the sender’s internal state. In this research, we asked whether there is a functional benefit of a high-sensitivity sensory system that picks up fear signals from even the lowest doses of fear in sweat. We found that humans actually communicate how much fear they are experiencing via their body odor. What is more, humans seemingly tag different fear doses as “fearful” or “disgusting” following an all-or-none principle. This ensures that the receivers of these fear odors are safe rather than sorry. Our research shows that social smells interact cross-modally with ambiguous visual information to exert their functions beneath the radar of conscious reporting, underlining smells’ stealth influence on our lives.

### Participants

Thirty-one right-handed, healthy female participants (mean age = 23.23 years, range = 18–38) provided written informed consent to take part in the study, which was approved by the University of Pennsylvania Institutional Review Board (Protocol No. 828758). We recruited female participants because of their tendency for superior olfaction (Brand & Millot, 2001) and because they typically show larger effects of olfactory-induced emotional contagion (de Groot et al., 2014; de Groot & Smeets, 2017), thus increasing the study’s effectiveness. Our estimated participant numbers were based on effect sizes obtained in comparable studies (summarized by de Groot & Smeets, 2017), in which 25 to 30 participants typically provided sufficient statistical power (80%) to detect a small-to-medium-sized effect (Hedges’s *g* = .36, 95% confidence interval [CI] = [.31, .41]) on comparable indirect measures of emotion, such as facial electromyography, face ratings, startle reflexes, and neural responses (α = .05; correlation among repeated measures: *r* = .8). Our sample size was also dependent on the amount of sweat pads available from our previous study (de Groot et al., 2020), with 12 participants (senders) estimated to provide enough material for 32 receivers. All participants reported being fluent in English and that they had normal or corrected-to-normal vision; no abnormal neurological conditions, head trauma, or brain lesions; no significant olfactory impairment; and no allergies; all reported that they were nonsmokers, not pregnant, and not taking medication that could impact the study.

### Materials

#### Odor stimuli

We used fear and neutral sweat samples from 36 senders enrolled in a previous study (de Groot et al., 2020). Starting 2 days before the donation session, these participants followed strict criteria based on prior experiments (Kamiloglu et al., 2018; Zhou & Chen, 2009) to avoid contamination of their sweat samples (e.g., the consumption of garlic and the use of fragranced hygiene products were prohibited). On the test day, they watched 30 min of pilot-tested fear clips (i.e., “Vicious,” “Cop Cam,” “100% Organic,” “Mr. Creak,” “Night Night Nancy,” “Blair Witch Project,” and “The Binding Box”; available at https://osf.io/4dtqb/) and a calming wildlife documentary (the BBC’s “Yellowstone: Autumn”), counterbalanced and separated by a 10-min break, while we measured their subjective emotional experience and physiological responses (heart rate, skin conductance, and respiratory rate). To assign senders to three categories of fear intensity (low, medium, and high), we used a multivariate approach that combined participants’ physiological responses and subjective ratings. Specifically, partial-least-squares discriminant analysis produced a regression equation that weighted the unique contribution of the subjective and physiological variables to yield the best classification of fear (vs. neutral) responses (100% classification accuracy; for the regression equation, and for an absolute ranking of variables, see the Supplemental Material). Multiplying these regression weights with participants’ raw scores (obtained in the fear condition) resulted in a composite fear score. On the basis of this score, we ranked 36 fear-induced participants and divided them into three groups (*n* = 12). Classification into the low-, medium-, and high-fear groups occurred well above chance (83% vs. 33%), as shown by unbiased leave-one-subject-out cross-validation (for more details, see de Groot et al., 2020).

For each odor category (low fear, medium fear, high fear, neutral), sweat pads were pooled across six senders (each contributing 12.5 cm^2^) to attenuate interindividual differences in sweat production, causing stimuli to regress in fear level to the mean of each category (low, medium, high). Because photoionization-detector recordings showed signs of sweat-stimulus decay on the second use (i.e., the sweat emitted significantly fewer volatiles; see de Groot et al., 2020), each receiver was presented with fresh samples. Odors were delivered using a custom-built, four-channel, computer-controlled olfactometer. Sweat pads (75 cm^2^) were distributed over four widemouthed 60-ml amber bottles (Fisherbrand, Waltham, MA). The bottles’ polyvinyl-lined caps contained holes for Versilon SE-200 inert tubing (1/8-in. inner diameter, 1/4-in. outer diameter; US Plastic, Lima, OH), so that medical-grade room air could carry the odor to the nasal endpiece that was connected to the participant’s nose (akin to the way air circulation in the real world would carry odorants from the body or clothing to your nose). A MATLAB script (The MathWorks, Natick, MA) triggered the randomized opening of one of four odor valves and their connected odor channel (mean intertrial time: 12.5 s). The olfactometer was equipped with two independent mass-flow controllers (Alicat Scientific, Tucson, AZ) that kept a constant airflow (3.2 liters per minute). During stimulus presentation, 95% of air (3.04 liters per minute) traveled over the sweat pad (odor channel), while 5% followed a separate air channel, devised to wash out residual odor between stimulus presentations.

#### Face stimuli

We used images of two male Caucasian actors from the Radboud Face Database (IDs 28 and 33) displaying fearful and disgusted faces (Langner et al., 2010). Using Photoshop, we converted these stimuli to gray scale and removed features exterior to facial musculature (hair, ears, and background). We then morphed these processed images using FantaMorph software (Version 5.4.8, Abrosoft, 2016) to create a set of ambiguous faces. Our final set of stimuli consisted of anchor images (100% disgust, 0% fear; 0% disgust, 100% fear) and morphs containing a mixture of disgust and fear (in 1% increments, from 1% to 99%). We perceptually tailored the stimuli, calculating each participant’s point of subject equality (PSE; the most ambiguous face) without olfactory cues prior to testing. We then selected six morphs surrounding this point (−15%, −9%, −3%, +3%, +6%, and +15% fear) for each actor for the eventual scanning session. For example, if a participant’s PSE for a given actor was a face originally morphed with 46% fear and 54% disgust, the eventual morphs used be 31% fear, 37% fear, 43% fear, 49% fear, 55% fear, and 61% fear.

#### Pilot test: artificial sweat and morph-task performance

**Fig. 2.**
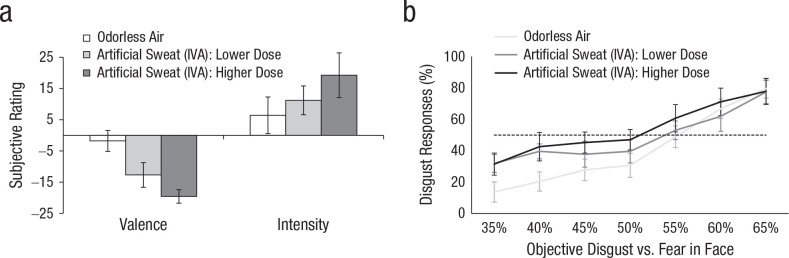
Results from the pilot study: mean (a) subjective ratings of odor pleasantness and intensity and (b) disgust ratings of faces morphed between disgust and fear. In each graph, ratings are shown as a function of odor: a higher dose of artificial sweat (isovaleric acid [IVA]), a lower IVA dose, and odorless air (control). In (b), the dashed line indicates the point of subjective equality, at which 50% of the responses are “fear” and 50% are “disgust.” Error bars represent ±1 *SE*.

We pilot-tested whether our morph task was subject to olfactory influence. Nine female participants (mean age = 21.56 years, range = 18–26) were asked to classify the facial expression of two male actors (IDs 28 and 33) from the Radboud Face Database. The actors’ expressions were morphed between disgust and fear (35%–65%), and participants had to classify each expression as “disgust” or “fear” while smelling two different doses of artificial sweat smell (isovaleric acid [IVA]) and odorless air. Participants completed six blocks of 21 trials (unique combinations of seven morphs and three odors). Prior to the morph task, the level of IVA was intensity calibrated per participant by modifying the olfactometer’s airflow passing through the odor bottle (vs. a separate air channel) and, if necessary, by changing the IVA concentration. This way, we created a subjectively perceived weaker IVA stimulus (lower dose: *M* = 0.014% IVA in mineral oil, range = 0.01%–0.05%) and a stronger IVA stimulus (higher dose: *M* = 0.3%, range = 0.1%–1%; Fig. 2).

A repeated measures analysis of variance (ANOVA) showed a significant main effect of odor, *F*(2, 16) = 5.48, *p* = .015, η^2^ = .04, 90% CI = [.02, .61], and of morph level, *F*(6, 48) = 15.01, *p* < .001, η^2^ = .45, 90% CI = [.46, .71]. Although the number of morphs identified as “disgust” were not differentially impacted by the lower and higher IVA dose, *t*(16) = 1.30, *p* = .213, *d* = 0.38, 95% CI = [−0.11, 0.87] (Holm-Bonferroni corrected), when compared with odorless air, both doses of artificial sweat biased participants to see more disgust across the spectrum of fear–disgust morphs, *t*(16) = 3.05, *p* = .008, *d* = 0.97, 95% CI = [0.08, 1.86]. We expected this disgust perception bias as induced by artificial sweat to be reversed for human sweat carrying fear-related information capable of inducing a greater fear perception bias.

### Procedure

**Fig. 3.**
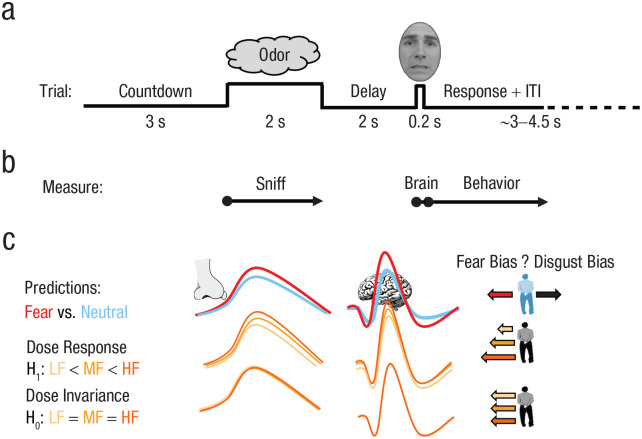
Experimental paradigm and predictions. At the start of the experiment, participants provided odor ratings (pleasantness, intensity) and were asked to discriminate between odors. This was followed by the main paradigm (a), in which each morph-task trial began with a 3-s countdown (3, 2, 1) and was followed by a sniff cue, during which the odor was presented. After a short delay, participants saw a face and classified it as either “fear” or “disgust.” The recording timeline (b) shows the main outcome variables: sniff (duration, volume), brain activation, and behavior (bias in judging ambiguous faces). The schematic of expected effects (c) is divided into categorical effects (fear odor [red line] vs. neural odor [blue line]), forming a replication of prior research, and fear-dose effects showing either a linear dose-response function (alternative hypothesis [H_1_]) or dose-invariance (null hypothesis [H_0_])—low fear (LF [yellow line]) vs. medium fear (MF [light orange line]) vs. high fear (HF [dark orange line]). Higher amplitudes (lines) reflect an expected stronger response (sniffing, brain activity). In the righthand column, larger arrows to the left reflect an expected greater fear bias on the face-morph task. ITI = intertrial interval.

On the test day, frozen sweat pads were removed from the freezer (−80° C) approximately 45 min prior to olfactometer delivery in the scanner. Participants were first asked to rate the intensity and pleasantness of all four odors (neutral, low fear, medium fear, high fear). This was followed by a discrimination task, which consisted of 12 trials of a two-alternative forced-choice reminder task (Van Hout et al., 2011). On each trial, participants first smelled a reference odor, after which they were presented with two test odors, one of which was the reference odor. Participants had to select the reference odor from the test odors (50% chance). These psychometric tasks were conducted to ensure that our neural and behavioral effects were not driven by conscious awareness of hedonic differences or by the explicit ability to discriminate the odors. We conducted these tests at the beginning of the experiment to ensure maximal odorant delivery so that our results would not be confounded either by lack of odorant delivery or by psychophysiological effects such as adaptation or habituation. After the psychometric tasks, our main paradigm (Fig. 3a) employed a face-morph task. In this procedure, participants had to decide that an emotionally ambiguous face displayed fear or disgust. A few seconds before the face appeared, participants had to make a brief sniff to detect a very small quantity of odor that they were told would be added to the air stream, which could be different each time. Participants were told about the nature of the odors and their expected influence on face ratings only during the debriefing.

Participants had two no-odor practice blocks (total: eight trials) using the anchored faces (100% fear or 100% disgust) prior to beginning the main task (cf. Mujica-Parodi et al., 2009). The main task consisted of 10 blocks, each comprising 24 randomized trials presenting unique combinations of morph level (six) and odor (four). Breaks between blocks were self-paced. The whole procedure was double blind.

### Statistical analysis

#### Data exclusion

Similar to procedures preregistered in other fMRI work (Botvinik-Nezer et al., 2019), four participants’ data were fully excluded because of a high (> 20%) rate of missing behavioral responses (*M* = 25.11% invalid responses: 0.21% of responses were too fast, 7.81% were too slow as per our criteria—see the Morph Task subsection under Data Analysis—and 17.09% were missing altogether). We verified that invalid responses occurred randomly across fMRI runs, morph levels, and odors (see Fig. S3 in the Supplemental Material), which could indicate participants’ general fatigue, boredom, or lack of confidence in their responses. In addition, one run for three participants was excluded because of a technical error in the procedure, and three trials across two participants were excluded from fMRI analysis because they missed the sniff cue. Additionally, odor-intensity, valence, and discrimination ratings were missing for one of the participants. One other participant dropped out in the middle of the study, and her data were not included in the analyses. This left 27 participants for the main analyses and 26 for odor ratings.

In a separate study (de Groot et al., 2020), we had used a photoionization detector to quantify volatile molecules given off by sweat, and we observed that repeated olfactometer-based odor presentations changed the neutral and fear sweat’s quantity of volatiles emitted according to a biexponential decay function. Pilot tests using a photoionization detector indicated that volatile quantity waned to almost negligible levels after approximately 30 presentations of that odor (see Fig. 1d, as well as Fig. S2 in the Supplemental Material). Therefore, we took a behavior-driven Bayesian-analysis approach to discern whether the predicted categorical effects (fear sweat causing more morphs to be identified as fearful than neutral) were eradicated in the second of two experiment halves (after 30 presentations of each odor, i.e., after 120 trials). Indeed, a Bayesian paired-samples *t* test on morph ratings showed strong evidence (Bayes factor favoring the alternative over the null hypothesis, or BF_10_ = 31.07) for a categorical difference between fear odor and neutral odor in the first half of the experiment, whereas this evidence dropped to anecdotal levels in the second half (BF_10_ = 1.42). On the basis of these findings, and to be able to test fear-dose-response functions, we used the data from the first five blocks for all subsequent analyses in which the focus was on dose-response relations. This was the first study in which the volatility of human sweat molecules was quantified as a function of time. It also confirmed that fear sweat does give off volatile molecules that can travel through an olfactometer air stream and reach a participant’s nose.

#### Data analysis

##### Psychometric tasks

Subjective ratings of odor intensity and pleasantness were submitted to a priori contrasts (a repeated measures ANOVA) comparing air with odor and comparing odors (neutral sweat and low-, medium-, and high-fear sweat). Odor-discrimination scores were compared with chance probability (.5) with a nonparametric one-sample *t* test.

##### Morph task

We followed morph-task data-exclusion procedures documented in comparable prior research (Mujica-Parodi et al., 2009) and discarded all responses shorter than 200 ms and exceeding 2,500 ms. There were two main variables of interest: participants’ average proportion of fear responses (calculated per odor and per morph) and participants’ PSEs (i.e., the actual morph step yielding 50:50 fear:disgust responses). The latter measure was used because prior research has shown that ambiguous faces are most susceptible to olfactory influence (Zhou & Chen, 2009). Therefore, the former measure could be dampened by including responses to less ambiguous faces. We estimated odor-dependent shifts in these most ambiguous points by fitting each participant’s data with a sigmoid function:

$$\begin{array}{c} \mathrm{Number}\;\mathrm{of}\;\mathrm{face}\;\mathrm{morphs}\;\mathrm{categorized}\;\mathrm{as}\;“\mathrm{fear}”\;(y)\;=\;\\ a\;+\;\frac{b}{1\;+\;e^{\left(-c\;\times \;(x\;-\;d)\right)}}. \end{array}$$

In this function, *y* represents the proportion of fear responses, *x* represents the morphing step, and coefficients *a* through *d* reflect the *y*-offset, height, slope, and inflection point of the curve, respectively. Using MATLAB’s curve-fitting toolbox, we set coefficients *a* and *b* to 0 and 100 to anchor the lower and upper asymptotes for the sigmoid curve (producing high *R*^2^s, *Mdn*: 0.86, *SE* = 0.02); *c* and *d* were estimated. Then we calculated each participant’s PSE (i.e., the point at which 50% of the response was fear) for each odor condition:

$$\mathrm{Estimated}\;\mathrm{PSE}\;(x)\;=\;-\;\frac{\ln\left(\frac{a\;+\;b\;-\;50}{50\;-\;a}\right)}{c}\;+\;d.$$

In this function, “ln” refers to the natural logarithm. With coefficient *a* (*y*-offset) being 0 and *b* (height) being 100, the numerator simplifies to 1. Then, a repeated measures ANOVA was carried out over average morph responses and PSEs, comparing neutral odor with fear odor (averaged across intensities) to test a categorical effect and comparing among fear-odor intensities to test dose responses.

##### Physiological recording and analysis

Physiological monitoring was conducted through a Powerlab (AD-Instruments, Sydney, Australia). Participants’ respiration was recorded using an abdominal breathing belt (TSD221-MRI, Biopac Systems, Goleta, CA), and cardiac responses were measured using a custom magnetic-resonance-safe pulse transducer (ADInstruments). Physiological data were analyzed after band-pass filtering the raw signal (respiratory rate: 0.05–50 Hz; heart rate: 0.5–50 Hz) to remove drift and artifacts.

##### fMRI acquisition parameters

MRI data were collected with a 3T Siemens Prisma scanner equipped with a 64-channel head coil. A T1-weighted magnetization-prepared rapid-acquisition gradient-echo (MPRAGE) structural scan was acquired at the beginning of the experiment (repetition time [TR] = 2,200 ms, echo time [TE] = 4.67 ms, inversion time = 900 ms, voxel size = 1 mm^3^, matrix size = 192 × 256 × 160 voxels). An interleaved T2*-weighted echoplanar image (EPI) sequence was used to collect blood-oxygen-level-dependent volumes (flip angle = 80°, 56 slices, voxel size = 2 mm^3^, no gap, matrix size = 104 × 104 voxels, field of view = 208 × 208 mm, TR = 2,000 ms, TE = 22 ms, multiband = 2, acquisition angle = 15° tilt from anterior commissure-posterior commissure, partial Fourier = 6/8).

##### fMRI preprocessing

We conducted fMRI preprocessing and analysis in MATLAB using Statistical Parametric Mapping (SPM) 12 software (Wellcome Trust Centre for Neuroimaging, London, UK) unless otherwise stated. We corrected for head motion by realigning EPI volumes to the first acquired image. The T1 structural images were then coregistered to the mean whole-brain EPI. We segmented anatomical images using the six-tissue probability map in SPM and applied the deformation fields to functional images for normalization into Montreal Neurological Institute (MNI) space. The resulting volumes were spatially smoothed with a 6-mm full-width half-maximum Gaussian kernel.

##### fMRI analysis

We conducted first-level analysis on concatenated fMRI runs at the within-participant level. This modeled 24 unique odor–face combinations at the time of face onset. Nuisance regressors included six movement parameters; sniff trace was derived from the respiratory effort band signal—smoothed (250-ms moving window), high-pass filtered (cutoff, 0.05 Hz), normalized (subtracting the mean, dividing by *SD*), down-sampled (0.5 Hz)—and motion scrubbing (framewise displacement threshold of 1.3 mm (see Siegel et al., 2014). For our primary whole-brain contrasts, we used a *t* test comparing trials in which activations in response to neutral odor (all faces) were lower than those in response to fear odor (all faces) with PSE as a covariate. This allowed us to look at effects collapsed across all odors. We examined whole-brain results at two levels of correction. Our more stringent, peak-level threshold applied whole-brain family-wise error (FWE) correction (*p*_FWE_ < .05). However, as this can be considered too strict a threshold, with potential for false negatives (Eklund et al., 2016), we also looked at whole-brain results (uncorrected *p* < .001) that surpassed cluster-level correction (*p*_FWE-cluster_ < .05).

For our region-of-interest (ROI) analysis, we produced first-level contrasts comparing the three levels of fear-odor intensity (low, medium, high; neutral was subtracted). To test the influence of fear-odor intensity and face morphs on our ROIs, we extracted beta weights from the amygdala (anatomically defined) and the fusiform gyrus (functionally defined). Because most ROI-based parcellations fail to segregate the piriform cortex from the amygdala, we defined these ROIs with reference to a human neuroanatomical atlas (Mai et al., 2004), oriented in standard MNI space, that has been used in previous olfactory studies (e.g., Howard & Gottfried, 2014). Because the fusiform gyrus encompasses a relatively large section of the temporal lobe, and previously observed clusters do not span the entire region (Prehn-Kristensen et al., 2009; Wudarczyk et al., 2016), we specified this ROI using a leave-one-subject-out procedure (as instructed by Esterman et al., 2010). Second-level analyses (fear odor > neutral odor) were run for all but one subject; voxels within the fusiform gyrus surpassing *p* < .001 (uncorrected) were then used as a mask to extract beta values from the remaining participant (see Esterman et al., 2010). This provided us with unbiased beta weights. Any data used to define an ROI were not included in the beta values extracted, thus avoiding circular analyses (Kriegeskorte et al., 2009). The right fusiform gyrus did not generate clusters across all iterations. Specifically, no cluster could be defined for four of the participants, and these participants were omitted from the ROI analysis of the right fusiform gyrus. All beta weights were submitted to repeated measures ANOVAs across fear-odor intensities.

##### Bayesian analysis

Traditional null-hypothesis significance testing was complemented by Bayesian hypothesis testing to quantify the relative strength of evidence for the null hypothesis (H_0_; low fear = medium fear = high fear). This was compared with the alternative dose-response hypothesis (H_1_; low fear < medium fear < high fear), which was tested as an unconstrained hypothesis (low fear ≠ medium fear ≠ high fear) using JASP software (JASP Team, 2020; for a data-supported rationale, see Fig. S6 and Table S1 in the Supplemental Material). In JASP, we performed Bayesian repeated measures ANOVAs with default (noninformative) prior settings (*r*-scale fixed effects = .5; *r*-scale random effects = 1). We report the Bayes factor (BF) and the proportional error estimate on the BF. The subscripts on BFs refer to the models compared. Accordingly, the BF for the null relative to the alternative hypothesis is denoted BF_01_. In our interpretation of the BFs, we follow the classification by Lee and Wagenmakers (2013): BF_01_ from 1 to 3 indicates anecdotal evidence for H_0_ over H_1_, from 3 to 10 indicates moderate evidence, from 10 to 30 indicates strong evidence, from 30 to 100 indicates very strong evidence, and greater than 100 indicates extreme evidence.

## Results

Having established that the degree of fear scales behaviorally with the total amount of fear sweat induced in the sender participants, we next focused on whether different levels of fear sweat would modulate physiological, behavioral, and neural responses in the receiver participants (Fig. 3).

**Fig. 4.**
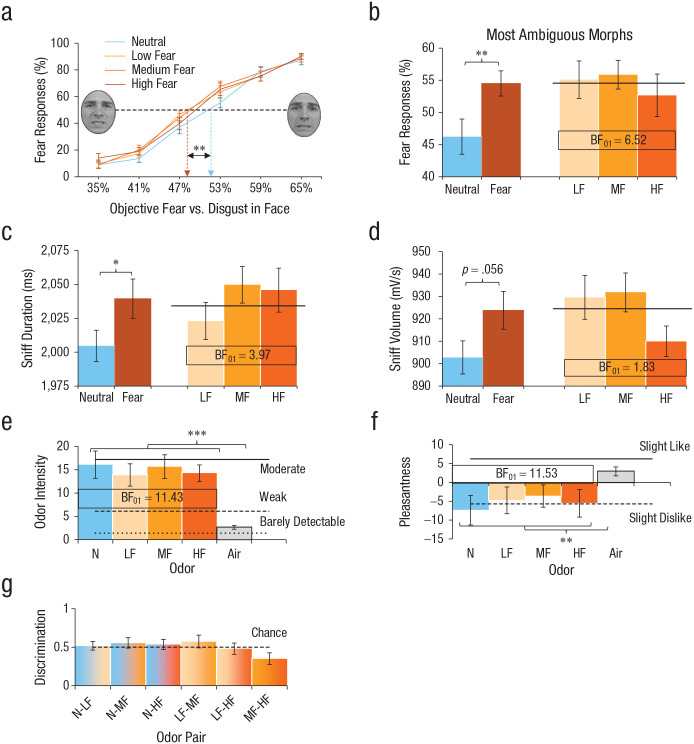
Effects of sweat odor (low fear [LF], medium fear [MF], high fear [HF], and neutral [N]) on mean (a) fear responses on the whole-face-morph task, (b) fear responses on morphs maximally ambiguous between disgust and fear, (c) sniff duration, (d) sniff volume, (e) odor-intensity ratings, (f) odor-pleasantness ratings, and (g) ability to discriminate odor pairs. The dashed line in (a) indicates the point of subjective equality, at which 50% of the responses are “fear” and 50% are “disgust.” The brown bar in (b), (c), and (d) is the average of all three fear-sweat levels used to test for categorical effects (fear vs. neutral). The horizontal line in (b), (c), and (d) indicates the average of the three fear-sweat levels. Asterisks indicate significant differences between conditions (*p < .05, **p < .01, ***p < .001). Bayes factor BF_01_ indicates evidence in favor of the null hypothesis (dose invariance: LF = MF = HF) over the alternative hypothesis (linear effect: LF < MF < HF). Error bars represent ±1 *SE*.

At the behavioral level, we tested the hypothesis that receivers exposed to higher levels of fear sweat would systematically perceive higher amounts of fear in the more ambiguous faces (Mujica-Parodi et al., 2009; Zhou & Chen, 2009). The prediction was that compared with smelling neutral sweat, smelling each level of fear sweat—low fear, medium fear, or high fear—would bias face perception in the direction of fear rather than disgust, that is, face morphs containing greater proportions of veridical disgust would be perceived as fearful. The shape of this function was expected to be fear-dose-dependent (H_1_) or, alternatively, fear-dose-invariant (flat line; H_0_) and was evaluated using Bayesian statistical approaches. Interestingly, although we found that each level of fear sweat induced a leftward (fear) bias in the perception of facial expressions versus neutral sweat, *F*(1, 26) = 13.44, *p* = .001, Cohen’s *d* = 0.51, 95% CI = [0.22, 0.81] (Fig. 4a), Bayesian analysis on fear bias revealed moderate evidence for fear-dose-invariant effects (BF favoring H_0_ over H_1_: BF_01_ = 8.39 ± 1.0%). Post hoc tests comparing each fear sweat level to neutral sweat showed that sniffing fear sweat induced a medium-sized shift toward the disgust faces being perceived as more fearful—low fear vs. neutral: *F*(1, 26) = 8.34, *p* = .008, *d* = 0.46, 95% CI = [0.06, 0.87]; medium fear vs. neutral: *F*(1, 26) = 7.70, *p* = .010, *d* = 0.54, 95% CI = [0.19, 0.89]; high fear vs. neutral: *F*(1, 26) = 7.43, *p* = .011, *d* = 0.45, 95% CI = [0.16, 0.74]). Notably, the most ambiguous morphs were most susceptible to the influence of fear sweat, *F*(1, 26) = 13.36, *p* = .001, *d* = 0.65, 95% CI = [0.26, 1.04], dose invariance: BF_01_ = 6.52 ± 1.0% (Fig. 4b).

In a parallel analysis of the physiological data, we predicted that compared with smelling neutral sweat, smelling each level of fear sweat (low, medium, high) would increase the speed and volume of the sniff response, representing a sign of adaptive sensory vigilance (de Groot et al., 2012). While each level of fear sweat induced a small fear-specific increase in sniffing responses—duration: *F*(1, 26) = 4.71, *p* = .039, *d* = 0.22, 95% CI = [0.17, 0.41]; volume: *F*(1, 26) = 4.01, *p* = .056, *d* = 0.39, 95% CI = [0.22, 0.57]—moderate evidence was obtained for dose invariance on sniff duration (BF_01_ = 3.97 ± 0.9%), whereas evidence for dose invariance on sniff volume was only anecdotal (BF_01_ = 1.83 ± 0.8%) because of the high-fear-sweat drop in sniff volume (Figs. 4c and 4d). Of note, the effects of fear sweat on both behavior and physiology were observed despite the fact that all sweat stimuli were indistinguishable, equally intense (strong evidence: BF_01_ = 11.43 ± 0.7%) and equally pleasant (strong evidence: BF_01_ = 11.53 ± 0.7%; Figs. 4e–4g; see the results in the Supplemental Material). These seemingly paradoxical findings replicate those of prior research (de Groot et al., 2018; Mujica-Parodi et al., 2009) and may be intelligible from neurocognitive limitations in translating odor experiences into words (Olofsson & Gottfried, 2015).

**Fig. 5.**
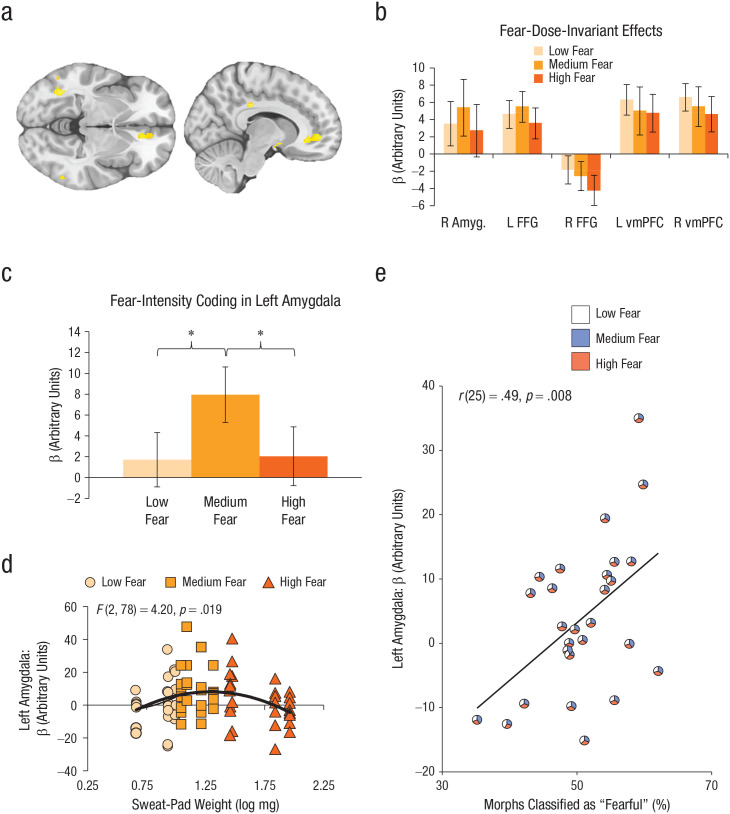
Results of whole-brain, region of interest (ROI), and exploratory analyses. The brain images (a) show activations from the whole-brain contrast (fear odor > neutral odor) in fusiform face gyrus (FFG) and ventromedial prefrontal cortex (vmPFC; uncorrected *p* < .001, 10-voxel threshold). Fear-dose-invariant effects (b; neutral odor was subtracted from each fear level) are shown for separate ROIs: right amygdala, left and right FFG, and left and right vmPFC. Fear-intensity coding (c; fear odors – neutral odors) is shown for the left amygdala. Left amygdala activity (d) is shown as a function of sweat-pad weight and presented fear-sweat dose. The curved line shows the best-fitting linear regression. The scatterplot (e) shows the correlation between left amygdala activity in response to fear sweat (low, medium, high) and the percentage of morphs identified as “fear” (vs. “disgust”). The diagonal line shows the best-fitting regression. Each individualized pie chart displays the relative contribution of low-fear, medium-fear, and high-fear odor trials classified as “fear” to overall fear responses on the morph task and left amygdala activity. Asterisks in (c) indicate significant differences between conditions (*p < .05). Error bars in (b) and (c) represent ±1 *SE*.

We next conducted a second-level fMRI analysis to demonstrate whole-brain responses to fear odor (averaged across intensities), in comparison with neutral sweat. We saw significantly increased activation for clusters in the FFG, *t*(25) = 5.04, *p*_FWE-cluster_ = .0496 (*x* = −52, *y* = −44, *z* = −16, 110 voxels) and the ventromedial prefrontal cortex (vmPFC), *t*(25) = 4.39, *p*_FWE-cluster_ = .042 (*x* = 10, *y* = 48, *z* = −2, 115 voxels; Fig. 5a). We subsequently tested whether our ROIs—amygdala and fusiform gyrus (Maier et al., 2019; Mujica-Parodi et al., 2009; Prehn-Kristensen et al., 2009; Wudarczyk et al., 2016)—showed dose-invariant (H_0_) or dose-dependent (H_1_) responses to fear amount in sweat, collapsed across all faces in the spectrum (Fig. 5b). The results showed that fear-sweat quantity was partially coded in the left amygdala, *F*(2, 52) = 3.86, *p* = .027, η^2^ = .13, 90% CI = [.01, .27] (BF_01_ = 0.35 ± 4.2%, where H_1_: low fear ≠ medium fear ≠ high fear).

Interestingly, exploratory post hoc contrasts on left amygdala activity revealed a significant quadratic relationship amongst fear odors, *F*(1, 26) = 6.54, *p* = .017, η^2^ = .20, 90% CI = [.02, .44] (Fig. 5c). Specifically, medium-fear odor (*M* = 7.95, *SD* = 13.82) elicited a greater amygdala response than low-fear odor (*M* = 1.71, *SD* = 13.53), *t*(26), *p* = .017, *d* = 0.46, 95% CI = [−0.07, 0.98], yet high-fear odor (*M* = 2.04, *SD* = 14.68) evoked a smaller amygdala response than medium-fear odor, *t*(26) = 2.34, *p* = .023, *d* = 0.41, 95% CI = [−0.04, 0.87]. A closer look at differences within fear-dose categories revealed that the highest amygdala responses were in fact evoked by a bandwidth of fear doses around the medium level, including high doses of low fear and low doses of high fear, *F*(2, 78) = 4.20, *p* = .019, *R*^2^ = .10, compared with doses falling outside of this bandwidth (Fig. 5d). Of note is that a significant linear association emerged between left amygdala activity and the percentage of face morphs (within subjects) being perceived as fearful across all levels of fear sweat, *r*(25) = .49, *p* = .009 (Fig. 5e). In contrast to these dose-varying responses, we found only dose-invariant responses in fMRI activation profiles in our other ROIs: right amygdala, *F*(2, 52) = 0.48, *p* = .619, η^2^ = .02, 90% CI = [.00, .09], BF_01_ = 6.42 ± 0.9%; left fusiform gyrus, *F*(2, 52) = 0.48, *p* = .620, η^2^ = .02, 90% CI = [.00, .09], BF_01_ = 6.36 ± 1.0%; and right fusiform gyrus, *F*(2, 44) = 0.58, *p* = .566, η^2^ = .03, 90% CI = [.00, .12], BF_01_ = 5.08 ± 0.9% (see Table S2 for additional correlations between ROIs and sniff variables).

In our ROI analysis, the right FFG appeared to demonstrate overall reduced activation across fear-sweat doses despite having observed a cluster of increased activation at uncorrected thresholds (Fig. 5a). This is likely due to unreliable fear-odor effects on the right FFG, evidenced by the lack of activation at a corrected threshold and inability of the leave-one-subject-out procedure to define a mask for four of the left-out participants (despite using an uncorrected threshold). Consequently, we were unable to draw strong conclusions regarding the right FFG. Given the lateralized findings in the previous literature, which has typically documented left FFG activation in response to fear sweat (Maier et al., 2019; Prehn-Kristensen et al., 2009; Wudarczyk et al., 2016), researchers should seek to further elucidate the lateralized nature of fear chemosignals on fusiform gyri in future work.

## Discussion

In the present study, we sought to establish whether humans are capable of communicating fear quantity from a sender to a receiver by their body odor. Our results show that all sweat stimuli (low fear, medium fear, high fear, neutral) were indistinguishable on the explicit level (Figs. 4e–g); at the same time, smelling different doses of fear in sweat caused receivers to partially inherit a sender’s fearful state at the behavioral, physiological, and neural levels. This unique attempt to titrate the smell of fear elicited two key findings. First, we found varying effects of fear-sweat doses on activity in the left amygdala. Second, we found moderate evidence for dose-invariant effects of fear sweat on face identification, sniff responses, and activity in the FFG. These combined findings suggest that the human sense of smell engages a binary on/off mechanism for identifying body odor as a fearful stimulus.

The most widespread finding across our behavioral, physiological, and neural measures (except the left amygdala) was a dose-invariant effect of sweat sampled during low, medium, and high fear. In the context of sweat-evoked fMRI responses, stable activation across different levels of fear sweat was found in the FFG. Such a profile would be consistent with the idea that these brain areas reflect the consequences of pattern completion to render any level of fear sweat (above a certain minimum) as conveying the same alarm message (i.e., fear) in a dose-invariant way. Considered in this framework, it is possible that each level of fear sweat transmitted by a sender, even at minute doses, activates high-affinity receptors early in the inhalation of a receiver, inducing fear-based behavior in the recipient (cf. Bolding & Franks, 2017, 2018). This *temporal winner-takes-all principle* could explain the functional benefit of a high-sensitivity system to elicit fear, ensuring that the receiver is safe rather than sorry.

The present study thus found moderate evidence for dose-invariant fear effects across behavioral measures, physiological measures, and most neural measures. At first glance, these findings seem to be at odds with the literature that has documented dose-dependent response curves for smells in animals ranging from fruit flies (Si et al., 2019) to humans (Cometto-Muñiz & Abraham, 2016). However, we identified a number of factors that could account for why dose-dependent effects did not emerge in our study. First, the range of stimulus concentrations used to chart dose-response curves typically spans a wide array of logarithmic steps (typically, 10^−1^ to 10^−6^ concentration; Cometto-Muñiz & Abraham, 2016; Si et al., 2019), whereas in our study, the high-fear-sweat stimulus was within only one log-concentration step from the low-fear-sweat stimulus. Thus, we may not have had sufficient dynamic range to establish a dose-dependent effect. Second, it is highly plausible that the strongest dose-dependent effects may emerge near the level of conscious detection (perithreshold; Cometto-Muñiz & Abraham, 2016), whereas all of our odor stimuli were indiscriminable (subthreshold). This possibility would have precluded our ability to identify changes if these were most robust at the point at which they were just noticeably different. However, we think the most likely explanation may simply be that dose-dependent effects are primarily instantiated at the more peripheral levels of the olfactory system, especially the olfactory bulb, an area that cannot be routinely imaged with fMRI methods. By comparison, mechanisms of concentration-normalization (e.g., Bolding & Franks, 2017; Roland et al., 2016) may transform the coding properties in downstream cortical areas (which are accessible via fMRI methods) such that concentration-dependent effects in fusiform gyrus, vmPFC, and amygdala are not observed.

Our second important finding revealed dose-dependent coding of fear quantity in the left amygdala. Although the left amygdala has previously been implicated in fear-odor processing (Maier et al., 2019; Mujica-Parodi et al., 2009), we found that this region could track fear-odor dose, albeit not linearly. Prior research using nonsocial smells has identified the amygdala as coding the quantity (vs. quality) of olfactory stimulation (Anderson et al., 2003), where other research found the amygdala responding more robustly to high-intensity odors than to low-intensity odors when these odors were unpleasant or pleasant, but not neutral (Winston et al., 2005). These earlier findings suggest that the amygdala codes the overall emotional content of olfactory stimuli. By comparison, in context of socially based fear odor, our results revealed a somewhat different profile. Here, a curvilinear relationship was identified between the evoked fMRI response in the left amygdala and the intensity of fear odor, with the greatest response occurring within a bandwidth including the highest doses of low-fear sweat, medium doses of medium-fear sweat, and the lowest doses of high-fear sweat. Notably, we found a significant linear association between left amygdala activity and face morphs being perceived as fearful (vs. disgusting).

Given the vital evolutionary function of social communication of fear, the demonstration of a curvilinear relationship to fear dose in sweat raises intriguing questions. At the level of encoding, highly fearful humans may prioritize their own safety over communicating fear (Susskind et al., 2008) so as not to give away our fear to harmful conspecifics or predators. Because we know that high-fear states lead to higher overall sweat production (de Groot et al., 2020), a greater volume of eccrine sweat may effectively dilute the fear-active contents in the apocrine portion (Parma et al., 2017). Alternatively, an increase in other (nonfear) odorants from the apocrine sweat glands, such as thiols and acids, could otherwise mask the fear signal (Parma et al., 2017). Either of these mechanisms could explain why decoders (receivers) did not show a linear increase in behavioral, neural, and physiological dose-response effects from medium- to high-fear sweat.

In our study, the olfactometer’s airstream delivered odor compounds to a participant’s nose, which could resemble the natural transportation of chemosignals in real-world circumstances. In real life, however, humans wear deodorants and perfume in order to mask body odor, in addition to layers of clothing that could prevent smells from impacting another person’s behavior. However, recent studies have shown that even under these circumstances, participants made consistent and reliable smell-based social judgments at typical social distances (Gaby & Zayas, 2017), and despite being masked (e.g., by cedarwood oil or clove smell), body odors could elicit the expected neural, behavioral, and physiological responses (Cecchetto et al., 2019; Endevelt-Shapira et al., 2018). These findings translated to a real-world setting: Dental students’ performance was negatively impacted by unperceivable, masked fear sweat (Singh et al., 2018). Taken together, these findings suggest ecological validity, but we are still far from knowing whether there is a universal code to these social smells, which odorant receptors are involved in picking them up, and what is the range of settings under which human smells can influence us.

The question of whether human body odor can shape social communication has been considered among the 125 most compelling multidisciplinary scientific challenges (Kennedy & Norman, 2005; cf. Wyatt, 2020, for a constructive critical view on human pheromone research). Here, we bring new evidence that despite the great chemical complexity of odor stimuli, different levels of fear sweat can be chemically anchored to their original categorical source in the environment (a fearful sender; Smeets et al., 2020) while adaptively modulating fear-specific neural, behavioral, and physiological responses in receivers. Our findings suggest that even though certain brain regions (the amygdala) code chemical concentrations of fear in a dose-varying manner within a certain bandwidth, humans tag different fear doses in sweat as fearful following an all-or-none principle. Achieving this form of stability and simplicity is a remarkable feature of the human olfactory system, especially given the rapid fluctuations in odorant quantity and quality. That these odors can exert their functions beneath our conscious awareness underlines the potent yet unrecognized influence smells may have on our lives.

## Supplemental Material

sj-docx-1-pss-10.1177_0956797620970548 – Supplemental material for Titrating the Smell of Fear: Initial Evidence for Dose-Invariant Behavioral, Physiological, and Neural Responses Click here for additional data file. Supplemental material, sj-docx-1-pss-10.1177_0956797620970548 for Titrating the Smell of Fear: Initial Evidence for Dose-Invariant Behavioral, Physiological, and Neural Responses by Jasper H. B. de Groot, Peter A. Kirk and Jay A. Gottfried in Psychological Science

## Transparency

*Action Editor:* Daniela Schiller

*Editor:* Patricia J. Bauer

*Author Contributions*

J. H. B. de Groot and P. A. Kirk share joint first authorship. J. H. B. de Groot conceived of the study and designed it in conjunction with P. A. Kirk and J. A. Gottfried. J. H. B. de Groot and P. A. Kirk collected the data, and all the authors analyzed the data. J. H. B. de Groot and J. A. Gottfried coordinated the study. J. H. B. de Groot and P. A. Kirk wrote the manuscript. All the authors provided critical revisions and approved the final version for submission.

**Open Practices:** All data, scripts, and materials have been made publicly available via OSF and can be accessed at https://osf.io/y76p2. The design and analysis plans for the experiments were not preregistered. This article has received the badges for Open Data and Open Materials. More information about the Open Practices badges can be found at http://www.psychologicalscience.org/publications/badges.
