# Supplementary material for: Titrating the Smell of Fear: Initial Evidence for Dose-Invariant Behavioral, Physiological, and Neural Responses
Source: Psychol Sci. 2021 Mar 22;32(4):558–72. doi: 10.1177/0956797620970548 (PMC8726592; doi:10.1177/0956797620970548)
Supplement: sj-docx-1-pss-10.1177_0956797620970548 – Supplemental material for Titrating the Smell of Fear: Initial Evidence for Dose-Invariant Behavioral, Physiological, and Neural Responses [file sj-docx-1-pss-10.1177_0956797620970548.docx]

**Supplementary Online Material**

***Titrating the Smell of Fear: Initial Evidence for Dose-Invariant Behavioral, Physiological, and Neural Responses***

**Senders: Multivariate classification into fear groups**

Before assigning 36 donors to groups of low, medium, and high fear intensity, PLS-DA’s classification function was used to produce a regression equation that weighted (using unstandardized regression coefficients) the unique contribution of each subjective and physiological variable to yield the best classification of fear (vs. neutral) responses (for an absolute ranking, see Figure 1S). Next, a *composite fear score* (CFS) was computed per subject by multiplying each weight by participants’ raw score during fear induction:

$$Composite Fear Score=-0.096+0.053 \times\left( Tonic Skin Conductance Level \right)+ 0.024 \times\left( Phasic Skin Conductance Response \right)+ 0.002 \times\left( Heart Rate \right)+0.012 \times\left( Respiratory Rate \right)+ 0.348 \times(Sweat pad weight) + 0.095 \times(Ratings: High Arousal-Negative valence) + 0.060 \times(Ratings: High Arousal-Positive Valence) - 0.071 \times(Ratings: Low Arousal-Negative Valence) - 0.080 \times(Ratings: Low Arousal-Positive Valence)$$

Subjects’ CFSs were then used to subdivide fear-induced donors into three groups (*n* = 12) of experienced fear intensity, namely low (*M* = 0.63, *SD* = 0.06), medium (*M* = 0.88, *SD* = 0.06), and high (*M* = 1.17, *SD* = 0.10) (for more information, see de Groot, Kirk, & Gottfried, 2020).

**Significant linear effects of target states**

To verify whether the classification of fear responders (*N* = 36) into the different

intensity groups was successful, we conducted a one-way ANOVA on self-reported feeling states and physiological responses with fear intensity group (low, medium, high) as factor. As expected, this analysis yielded significant linear increases from low, to medium, to high, in self-reported fear: *F*(1, 33) = 9.08, *p* = .005, η^2^ = .22, 90% CI [.04-.43], self-reported tension: *F*(1, 33) = 9.59, *p* = .004, η^2^ = .23, 90% CI [.05-.44], self-reported nervousness: *F*(1, 33) = 11.31, *p* = .002, η^2^ = .26, 90% CI [.07-.47], self-reported stress: *F*(1, 33) = 9.14, *p* = .005, η^2^ = .22, 90% CI [.04-.43], armpit sweat production: *F*(1, 33) = 8.39, *p* = .007, η^2^ = .20, 90% CI [.04-.41], and skin conductance level: *F*(1, 33) = 25.94, *p* < .001, η^2^ = .44, 90% CI [.21-.63] (**Figure 1**, main text). Neither were there differences in self-reported disgust, nor in anger, *F* < 1 (**Figure S1**).
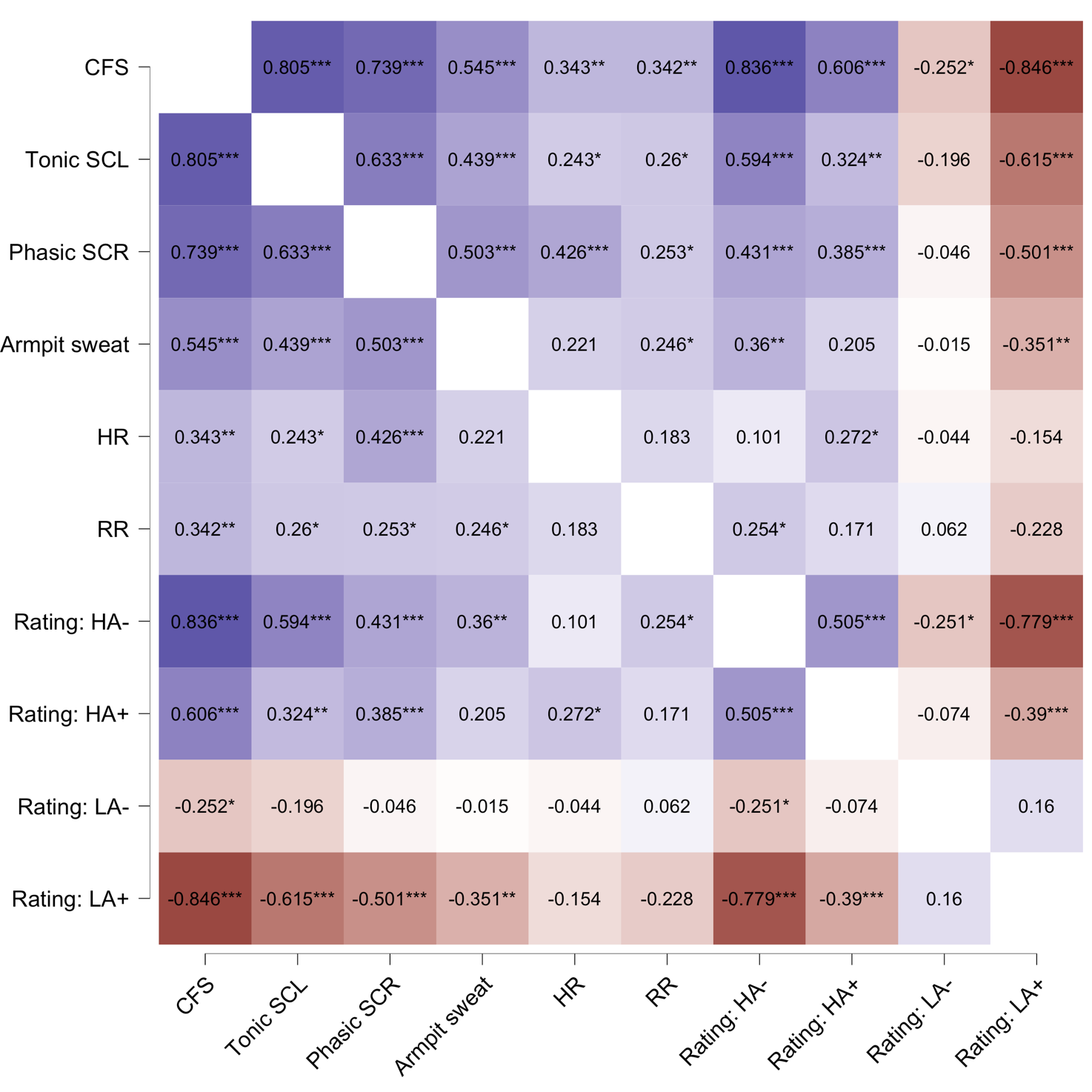


**A**

**Figure S1**. Data from “senders” (*N* = 36) providing fear and neutral sweat odor that was presented to another group of “receivers” in the main experiment. (**A**) Correlation heat map (Spearman’s ρ) between senders’ composite fear score (CFS), physiological responses and subjective ratings. Physiological variables: skin conductance level (SCL); skin conductance responses (SCR); quantity of armpit sweat; heart rate (HR); respiratory rate (RR). Subjective ratings: clustered on high arousal–negative valence items (HA−); high arousal–positive valence items (HA+); low arousal–negative valence items (LA-); and low arousal–positive valence items (LA+). For a rationale on self-report clustering, see de Groot et al. (2020). (**B**) Multivariate classification of senders’ fear responses using PLS-DA: Explanatory variables contributing most to the model based on ‘variable importance for the projection’ (VIP) scores (95% confidence intervals). Dark red bars: Higher values indicate a greater likelihood of a response being classified into “fear” vs. pleasant-neutral (blue bars). Figure adapted from de Groot et al. (2020). (**C**) Donors classified into the low (*n* = 12), medium (*n* = 12), and high fear (*n* = 12) intensity group did not differ in their experienced degree of disgust and anger during sweat collection.

**Exploring temporal relations between odorant quantity and face morph ratings**


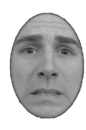

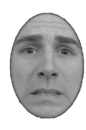


**D**


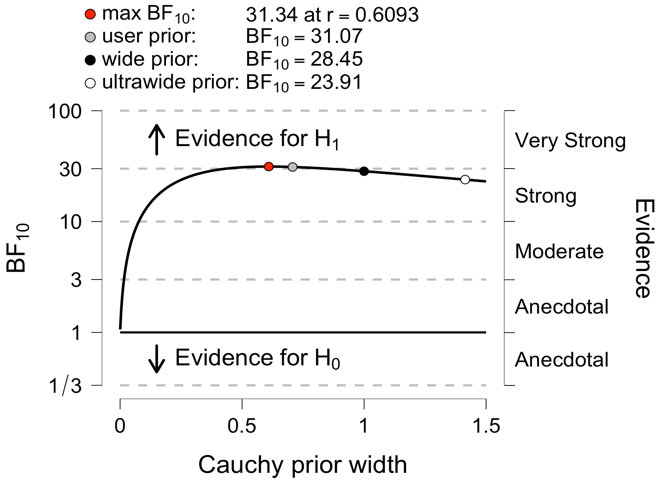

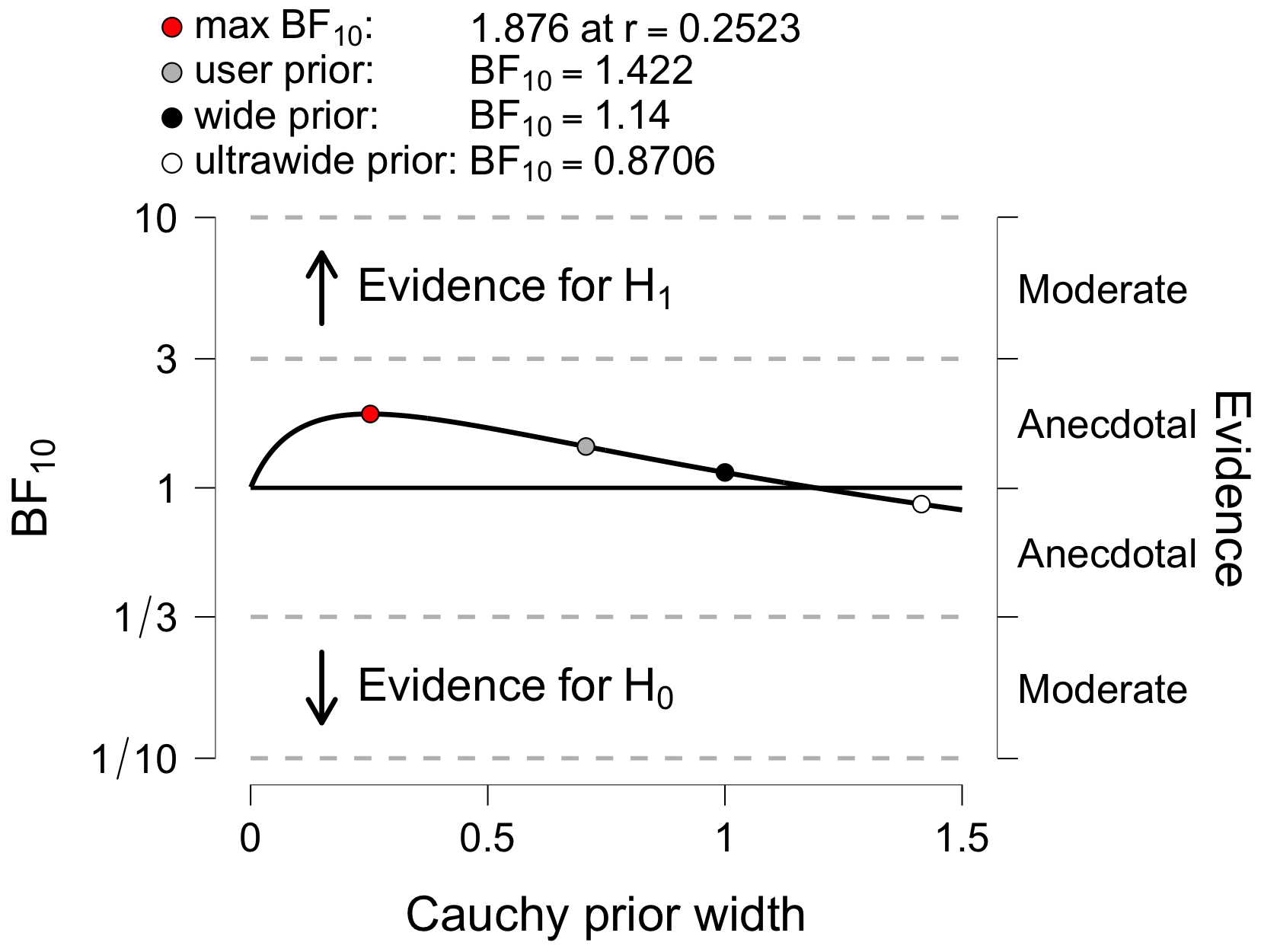


**C**

**Figure S2.** Exploring the temporal relation between sweat odorant quantity (tested separately on three samples using an olfactometer and photo-ionization detector; see: de Groot, Kirk, & Gottfried, 2020; Figure 1D) and participants’ fear bias on a face morph task (*N* = 31). We first report the absolute (**A**) and relative (**B**) quantity of odorant molecules (millivolt) given off by fear sweat and neutral sweat. In absolute terms, fear sweat (also higher in weight) emits more volatiles than neutral sweat, but it shows a bi-exponential decay function; after block 5, only a negligible amount of odorant is transported to the participant’s nose. In relative terms, the fear odor to neutral odor molecule ratio drops after an initial increase. Subsequent Bayesian analysis in JASP (JASP Team, 2020) of morph ratings split per experiment half (block 1-5, block 6-10) showed (**C**) strong evidence for more morphs identified as “fearful” for participants smelling fear (vs. neutral) sweat in the first five blocks, whereas (**D**) only anecdotal evidence for H_1_ was reported in the last five blocks. Arguably, this behavioral effect drop was caused by the delivery of *fewer* volatiles in *smaller* ratio between fear and neutral sweat and/or due to adaptation or habituation effects; this needs to be confirmed in future research. Note: 5 blocks equal ~30 min testing time.

**Performance on morph task during 2^nd^ experiment half: Frequentist approach**

A RM-ANOVA on morph ratings with odor (4 levels: neutral sweat, low fear sweat, medium fear sweat, high fear sweat) and morph level (6 levels: 35% fear, 41% fear, 47% fear, 53% fear, 59% fear, 65% fear) yielded only a significant main effect of morph level, *F*(5, 130) = 122.03, *p* < .001, η^2^ = .68, 90% CI [.77-.85], indicating that morphs containing more fear also elicited more fear responses. There was, however, neither a main effect of odor, *F*(3, 78) = .97, *p* = .409, η^2^ = .00, 90% CI [.00-.09], nor an interaction between odor and morph level, *F*(15, 390) = 1.12, *p* = .334, η^2^ = .00, 90% CI [.00-.04].

**Morph task: Brief analysis of invalid responses leading to participant exclusion**


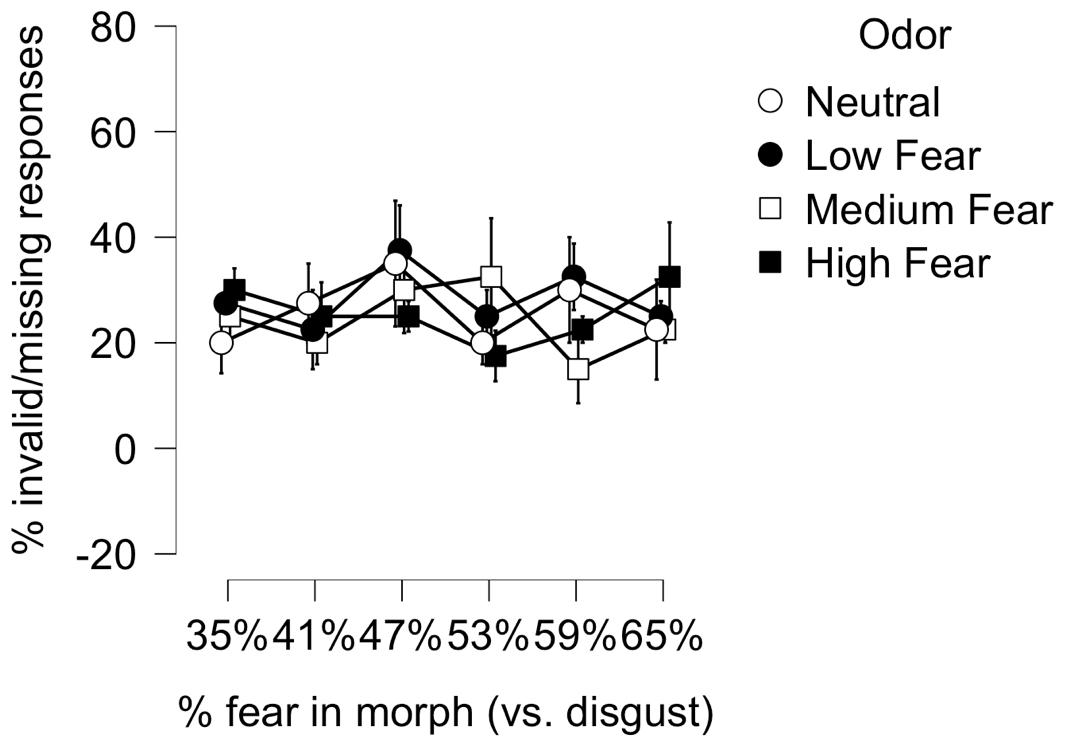


**Figure S3.** Distribution of missing/invalid responses on the morph task for the four excluded subjects (> 20% invalid data) showing a random pattern across morph levels and odors. Error bars ± 1 SE.

**Fear odors bias judgment of most ambiguous morphs**

First, a repeated measures ANOVA on morph ratings with odor (4 levels: neutral sweat, low fear sweat, medium fear sweat, high fear sweat) and morph level (6 levels: 35% fear, 41% fear, 47% fear, 53% fear, 59% fear, 65% fear) as within-subjects factors yielded a significant effect of odor, *F*(3, 78) = 3.50, *p* = .019, η^2^ = .12, 90% CI [.01-.22], and of morph level, *F*(5, 130) = 213.07, *p* < .001, η^2^ = .89, 90% CI [.86-.91].

To assess in more detail receivers’ shift in fear perception by fear odor (intensity),

we combined morphs into categories based on objective fear percentage (vs. disgust): “least fear” (35-41%), “most ambiguous” (47-53%), and “most fear” (59-65%).

As expected, fear odors most strongly biased perception in the fear (vs. disgust) direction for the *most ambiguous* morphs, *F*(1, 26) = 13.36, *p* = .001, *d* = .65, 95% CI [.26-1.04] (cf. Main text). Significant differences were found between neutral odor and low fear: *F*(1, 26) = 8.08, *p* = .009, *d* = .60, 95% CI [.14-1.06], neutral odor and medium fear: *F*(1, 26) = 11.27, *p* = .002, *d* = .74, 95% CI [.24-1.23], whereas the difference between neutral odor and high fear did not reach significance: *F*(1, 26) = 3.96, *p* = .057, *d* = .40, 95% CI [-.02-.83].

On the morphs containing the *least fear*, there was a significant linear contrast, indicating that a greater percentage of faces were identified as fearful as a function of increasing fear intensity, *F*(1, 26) = 5.25, *p* = .030, η^2^ = .17, 90% CI [.01-.40] (**Figure S4A**). There was neither a significant categorical difference between fear odor and neutral odor, *F*(1, 26) = 3.43, *p* = .075, *d* = .32, 95% CI [-.03-.68], nor between neutral odor and low fear: *F*(1, 26) = 1.00, *p* = .326, *d* = .20, 95% CI [-.01-.40], neutral odor and medium fear: *F*(1, 26) = 1.96, *p* = .174, d = .23, 95% CI [.04-.43]; yet, a significant difference did emerge between neutral odor and high fear: *F*(1, 26) = 4.99, *p* = .034, d =.41, 95% CI [.20-.63].

Judgment of morphs containing the **most fear** was clearly not impacted by odor, *F*s <1 (**Figure S4B**), indicating ceiling effects for faces containing more fear.

**Figure S4**. Percentage of faces identified as fearful by receivers as a function of odor (neutral, low fear, medium fear, high fear) per morph type: (**A**) Morphs containing relatively more disgust than fear (linear effect). (**B**) Morphs containing relatively more fear than disgust (ceiling effect).

**Categorical shift in Point of Subjective Equality (PSE)**

We conducted Sigmoid curve fitting in Matlab and ran a RM-ANOVA on

participants’ estimated Points of Subjective Equality (PSE), the morph step at which a decision between fear and disgust would be exactly 50-50. This analysis confirmed that face perception was biased toward fear in the fear odor condition, *F*(1, 26) = 9.59, *p* = .005, *d* = .51, 95% CI [.21-.80] (cf. Figure 4A, Main text). Notably, this *categorical* PSE shift (vs. neutral odor) was significant for low fear, *F*(1, 26) = 5.39, *p* = .028, *d* = .41, 95% CI [.08-.75], medium fear, *F*(1, 26) = 7.73, *p* = .010, *d* = .54, 95% CI [.12-.95], and high fear odor, *F*(1, 26) = 4.93, *p* = .035, *d* = .39, 95% CI [.05-.73]. Hence, it required less objective fear in a morphed face to arrive at a 50-50 fear-disgust decision in the context of fear odor.

Second, focusing on the dose-response function to fear odors, Bayesian analysis indicated that receivers’ behavioral responses to fear odors were more likely to be dose-invariant (H_0_) than linear (H_1_): BF_01_ = 8.16 ± 0.7%.

**Fear odor did not cause differences in reaction time on morph task**

Whilst we saw categorical bias in relation to face categorization, these

differences were not reflected in reaction time (RT) between neutral odor (*M* = 964.32 ms, *SD* = 184.96 ms) and fear odor (*M* = 957.41 ms, *SD* = 193.83 ms), *F* < 1. This was also true for RT differences between fear odor intensities, low (*M* = 947.16 ms, *SD* = 195.40 ms), medium (*M* = 955.38 ms, *SD* = 193.70 ms), and high (*M* = 969.70 ms, *SD* = 211.80 ms), *F* < 1. There was, however, a quadratic effect indicating that the highest RTs were found for the most ambiguous morphs, *F*(1, 26) = 21.47, *p* < .001, η^2^ = .45, 90% CI [.20-.66].

**Fear sweat-induced changes in breathing patterns**

At the physiological level, we supported our first prediction that all fear odors

would enhance breathing through the nose. That is, participants sniffed for longer during fear odor inhalation (*M* = 2039.47 ms, *SD* = 75.16) than during neutral odor inhalation (*M* = 2004.64 ms, *SD* = 60.19). Post hoc analyses comparing neutral odor to each fear odor showed that this effect was significant for medium fear, *F*(1, 26) = 5.62, *p* = .025, *d* = .71, 95% CI [.05-1.37], but neither for low fear (*F* < 1), nor for high fear odor, *F*(1, 26) = 3.09, *p* = .090, *d* = .59, 95% CI [-.12-1.30]. Sniff amplitude was unaffected, *F* < 1.

**Different neural responses to low fear (vs. neutral) odor despite equal weight**

The goal of this analysis was to verify whether receivers would show different

neural responses to two odors that were similar in sweat weight but that were different in the quality of the emotion condition (fear, neutral) under which they were produced. Our model compared brain activation in the context of low fear vs. neutral odor (collapsed across morphed faces). We saw significantly increased peak activation in the left fusiform gyrus (FFG: *t*(26) = 6.53, *p*_FWE_ = .032, at x = -40, y = -56, z = -8), and across a cluster in the ventral medial prefrontal cortex (vmPFC: *t*(26) = 5.01, *p*_FWE-cluster_ = .001, at x = 8, y = 38, z = 0; **Figure S5**). To corroborate these findings were not due to objective differences in sweat production, normalized sweat pad weights were included as nuisance regressor in the model, which did not alter the significance (FFG: *t*(25) = 6.49, *p*_FWE_ = .042, at x = -40, y = -56, z = -8; vmPFC: *t*(25) = 4.96, *p*_FWE-cluster_ = .002, at x = 8, y = 38, z = 0). By showing increased activation irrespective of volatile quantity, these results outline the greater engagement of FFG and vmPFC to *qualitatively* different molecules inherent to fear sweat.


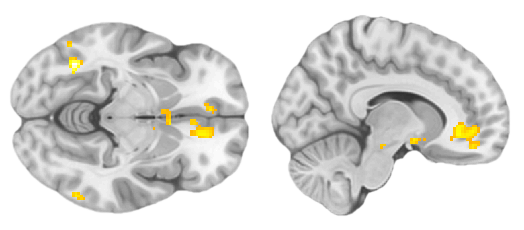


**Figure S5.** Whole-brain contrast (low fear > neutral odor) demonstrating increased activity in fusiform gyrus and vmPFC (p_uncorr._ < .001, 10 voxel threshold).

**Bayesian analysis: Fear dose-invariant responses (H_0_) vs. (un)constrained H_1_**

**Figure S6.** Posterior model probabilities showing the relatively likelihood of the hypotheses under consideration. These hypotheses compared dose-invariant to dose-dependent effects of low fear sweat (LF), medium fear sweat (MF), and high fear sweat (HF) on behavior, physiology, and neural responses. (**A, B**) Results showed that, generally, the preferred hypothesis is a fear dose-invariant hypothesis (H_0_: LF=MF=HF). Regarding the alternative hypothesis (H_1_), there was *no evidence* that an informative, constrained dose-dependent hypothesis (H_1c_: LF<MF<HF) performed better than the “fail safe” unconstrained hypothesis (H_1u_: LF,MF,HF), in which “any difference can be going on” (Hoijtink, Mulder, van Lissa, & Gu, 2019), and across the board, H_1u_ performed better than H_1c_. Thus, as a more plausible, inclusive model, and to arrive at more conservative Bayes Factors, we decided to report H_01u_ in the main text. Legend: “All morphs” refers to responses on the face morph task; “amb. morphs”: responses to the most ambiguous morphs (47%-53% fear); “PSE”: point of subjective equality; “FFG” = fusiform face gyrus.

**Table S1.** Bayes Factors indicate H_0_ generally outperforms H_1u_, which performs better or at least equal to H_1c_.

| Comparison: | All morph | Amb. morph | PSE | Sniff dur. | Sniff vol. | Left amyg. | Right amyg. | Left FFG | Right FFG |
| --- | --- | --- | --- | --- | --- | --- | --- | --- | --- |
| H_0_: LF=MF=HF  vs. H_1u_: LF,MF,HF | 12.05 | 9.18 | 11.47 | 5.91 | 1.84 | 0.35 | 6.44 | 5.43 | 6.89 |
| H_1c_: LF<MF<HF  vs. H_1u_: LF,MF,HF | 0.98 | 0.34 | 1.34 | 1.96 | 0.03 | 0.02 | 0.17 | 0.17 | 0.14 |

*Note.* These Bayes Factors were computed in R with the open-source package bain (version 0.2.4) which allows for testing constrained hypotheses (Hoijtink et al., 2019). The Bayes Factors in the main text were calculated with JASP software.

| **Table S2**  Bayesian Pearson Correlations between sniff variables (duration, volume) and regions of interest. All variables were averaged across fear sweat levels (low, medium, and high). | | | | | | | | | | | | | | | | | | | | | | |  |
| --- | --- | --- | --- | --- | --- | --- | --- | --- | --- | --- | --- | --- | --- | --- | --- | --- | --- | --- | --- | --- | --- | --- | --- |
| **Variable** |  | | **Sniff**  **duration** | | | | **Sniff**  **volume** | | **L**  **amygdala** | | | | **R**  **amygdala** | | | **L**  **FFG** | | **R**  **FFG** | | |  |  |  |
| Sniff duration |  | Pearson's *r* | |  | — |  | |  | |  |  |  | |  |  | |  | |  |  | |  | |
|  |  | BF₁₀ | |  | — |  | |  | |  |  |  | |  |  | |  | |  |  | |  | |
| Sniff volume |  | Pearson's *r* | |  | 0.558 | * | | — | |  |  |  | |  |  | |  | |  |  | |  | |
|  |  | BF₁₀ | |  | 18.464 |  | | — | |  |  |  | |  |  | |  | |  |  | |  | |
| L amygdala |  | Pearson's *r* | |  | -0.379 |  | | -0.243 | |  | — |  | |  |  | |  | |  |  | |  | |
|  |  | BF₁₀ | |  | 1.448 |  | | 0.486 | |  | — |  | |  |  | |  | |  |  | |  | |
| R amygdala |  | Pearson's *r* | |  | -0.459 |  | | -0.337 | |  | 0.890 | *** | | — |  | |  | |  |  | |  | |
|  |  | BF₁₀ | |  | 3.754 |  | | 0.973 | |  | 1.868e +7 |  | | — |  | |  | |  |  | |  | |
| L FFG |  | Pearson's *r* | |  | -0.138 |  | | -0.128 | |  | 0.535 | * | | 0.565 | * | | — | |  |  | |  | |
|  |  | BF₁₀ | |  | 0.299 |  | | 0.290 | |  | 12.183 |  | | 20.996 |  | | — | |  |  | |  | |
| R FFG |  | Pearson's *r* | |  | -0.310 |  | | -0.029 | |  | 0.357 |  | | 0.125 |  | | 0.228 | |  | — | |  | |
|  |  | BF₁₀ | |  | 0.689 |  | | 0.261 | |  | 0.967 |  | | 0.301 |  | | 0.432 | |  | — | |  | |
|  | | | | | | | | | | | | | | | | | | | | | | |  |
| * BF₁₀ > 10 (strong), ** BF₁₀ > 30 (very strong), *** BF₁₀ > 100 (extreme evidence for H_1_ vs. H_0_) | | | | | | | | | | | | | | | | | | | | | | |  |

**References**

de Groot, J. H. B., Kirk, P. A., & Gottfried, J. A. (2020). Encoding fear intensity in human sweat. *Philosophical Transactions of the Royal Society B*, *375*, 20190271. https://doi.org/10.1098/rstb.2019.0271

Hoijtink, H., Mulder, J., van Lissa, C., & Gu, X. (2019). A Tutorial on Testing Hypotheses Using the Bayes Factor. *Psychological Methods*. https://doi.org/10.1037/met0000201

JASP Team. (2020). JASP (Version 0.12.2) [Computer software]. Retrieved from https://jasp-stats.org/
